# Inhibitors of Transthyretin Amyloidosis: How to Rank Drug Candidates Using X-ray Crystallography Data

**DOI:** 10.3390/molecules29040895

**Published:** 2024-02-18

**Authors:** José P. Leite, Diogo Costa-Rodrigues, Luís Gales

**Affiliations:** 1 ICBAS—Instituto de Ciências Biomédicas Abel Salazar, Universidade do Porto, 4050-313 Porto, Portugal; 2 i3S—Instituto de Investigação e Inovação em Saúde, Universidade do Porto, 4200-135 Porto, Portugal; 3 IBMC—Instituto de Biologia Molecular e Celular, Universidade do Porto, 4200-135 Porto, Portugal

**Keywords:** transthyretin, amyloid inhibitor, kinetic stabilizer, X-ray crystallography

## Abstract

Amyloidosis is a group of protein misfolding diseases, which include spongiform encephalopathies, Alzheimer’s disease and transthyretin (TTR) amyloidosis; all of them are characterized by extracellular deposits of an insoluble fibrillar protein. TTR amyloidosis is a highly debilitating and life-threatening disease. Patients carry less stable TTR homotetramers that are prone to dissociation into non-native monomers, which in turn rapidly self-assemble into oligomers and, ultimately, amyloid fibrils. Liver transplantation to induce the production of wild-type TTR was the only therapeutic strategy until recently. A promising approach to ameliorate transthyretin (TTR) amyloidosis is based on the so-called TTR kinetic stabilizers. More than 1000 TTR stabilizers have already been tested by many research groups, but the diversity of experimental techniques and conditions used hampers an objective prioritization of the compounds. One of the most reliable and unambiguous techniques applied to determine the structures of the TTR/drug complexes is X-ray diffraction. Most of the potential inhibitors bind in the TTR channel and the crystal structures reveal the atomic details of the interaction between the protein and the compound. Here we suggest that the stabilization effect is associated with a compaction of the quaternary structure of the protein and propose a scoring function to rank drugs based on X-ray crystallography data.

## 1. Introduction

Transthyretin (TTR) is a plasma protein composed of four identical monomers assembled around a central channel where two thyroxine molecules can be accommodated. Several single-point mutations lead to a decrease in the stability of TTR, with the dissociation of the tetramer thought to be the initial and controlling step of the process of amyloid fibril formation associated with neurodegeneration [1]. Several experimental observations point to the displacement of the edge β-strands of the β-sandwich of the native monomer, exposing the penultimate β-strands [2,3,4], which allows novel β-sheet interaction to form between the TTR subunits, causing them to self-assemble into oligomers and finally to mature fibrils. Up until recently, the only therapy available was liver transplantation, which induces the production of the wild-type form of TTR (WT-TTR) by the liver and drastically reduces the concentration of the amyloidogenic TTR variant in the plasma. However, in the case of cardiac amyloidosis, deposition of WT-TTR aggregates frequently continues [5].

**Figure 1:**
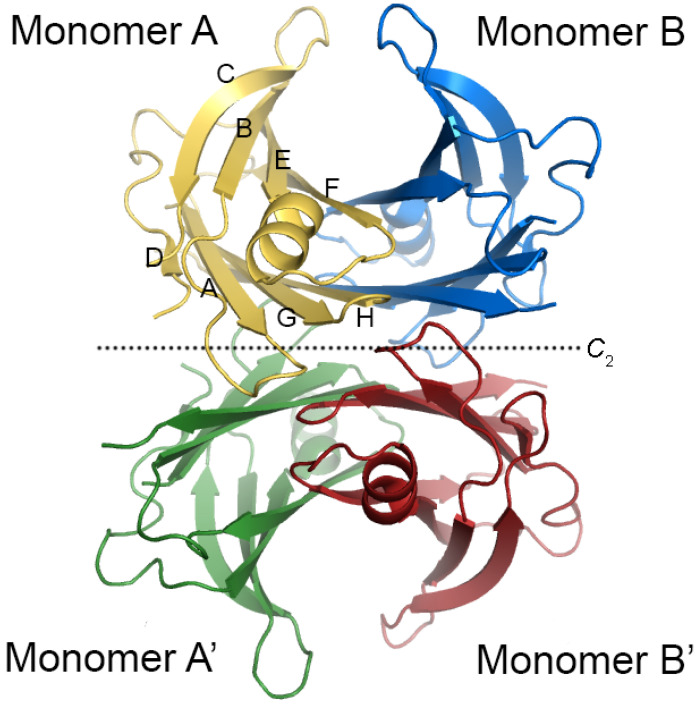
Crystal structure of wild-type transthyretin (WT-TTR). The protein is a homotetramer, and the monomeric subunits are colored differently. In one monomer, the eight β-strands are marked by ‘A’ through ‘H’ according to their sequential order from the *N*-terminal, showing the DAGH and CBEF β-sheets. The two dimers that form the tetrameric protein are related by a crystallographic 2-fold axis that bisects the hormone-binding channel. The AB/A’B’ is the energetically weaker interface that assembles around a central channel. Ligand binding to the central channel potentially contributes to the stabilization of the protein quaternary structure.

An emerging therapeutic strategy relies on the development of antisense oligonucleotides that inhibit the hepatic production of transthyretin (TTR). In this context, Tegsedi was approved by the European Commission for use in adults with stage one and two polyneuropathies [6]. More recently, the United States Food and Drug Administration (FDA) and Health Canada also approved its use for the treatment of the polyneuropathy of hereditary transthyretin-mediated amyloidosis (hATTR) in adults in the United States and Canada. An alternative strategy aims to discover compounds that preferentially bind to TTR in the plasma and stabilize the native structure of the protein, preventing dissociation and ultimately the production of amyloid aggregates [7,8]. Since TTR in the plasma is mostly free of thyroxine (T4) molecules [9] and there are other proteins that also transport T4 (thyroid-binding globulin and albumin) in the blood, the TTR T4 binding sites have been the preferential target of the so-called TTR kinetic stabilizers since it does not compromise the transport of T4. Those two binding sites are located at each side of the protein central channel, have a funnel shape and a hydrophobic character and contain three pairs of pockets with affinity towards halogen atoms [10]. Crucially, T4 binding sites are located at the interface between the two symmetry-related dimers AB/A’B’ (primed chains refer to those generated by the 2-fold crystallographic operation, see Figure 1), which is the energetically weaker dimeric interface of the protein [11]; the other interface AA’/BB’ is more stable. Thus, thyroxine-binding sites are likely the best TTR target to enhance protein stability.

Several hundreds of TTR kinetic stabilizers have already been tested [1,7,8]. Typically, the best compounds display two aromatic rings either directly fused or linked by a short spacer. Frequently, one of the rings has halogen substituents and preferentially binds deeply in the channel, and the other ring displays polar substituents that establish interactions with Lys15 or Glu54, both located at the mouth of the channel [1]. Interestingly, the stabilizers may also act as chaperones, enhancing TTR/amyloid-beta peptide (Aβ) interactions [12]. The amyloid beta peptide is associated with Alzheimer’s disease (AD) [13], and sequestration of this amyloid biomarker by TTR has a well-established role in neuroprotection in AD.

The evaluation of the TTR kinetic stabilizers usually starts with a series of in vitro experiments. The affinity of the drug candidates towards TTR is commonly tested using competition assays with the natural ligand T4; the drug effect on TTR stability is correlated with the extent of protein dissociation under semidenaturating conditions; in addition, the inhibition of fibril formation due to the presence of the compound is usually investigated using one of the many techniques available to follow the formation of amyloid aggregates, such as thioflavin-T fluorescence, light scattering or circular dichroism. A review of the literature shows that each research group has established its own protocols, which enables them to rank the drug candidates tested over the years. Merging the data collected by the different groups is more ambiguous due to the diversity of the experimental methods applied.

One of the more reliable and useful tools to study TTR amyloidosis is X-ray crystallography. It has revealed the atomic structure of the protein [14], how it interacts with the retinol-binding protein [15], the binding mode of T4 to TTR [10] and the structure of several mutants either with amyloidogenic or protective properties, and over the last few years, it has been used to implement the structure-based design of TTR kinetic stabilizers [16]. TTR yields quality X-ray diffracting crystals, and the determination of the structure, unlike most of the biochemical assays used to evaluate the ligands’ efficiency against TTR amyloidosis, is essentially unambiguous, usually performed by molecular replacement using a TTR high-resolution initial model. Thus, X-ray crystallography is a privileged source of experimental data that may be used to rank compounds evaluated by different research groups.

The models of the complexes TTR/kinetic stabilizers usually disclose subtle changes in the protein structure induced by ligand binding (alteration of residues’ side chain conformation, such as the rotation of Ser117 inducing the formation of a new intermonomer hydrogen bond, and stripping of structural water molecules) [16] and reveal the protein–ligand interactions. Based on this structural information, new compounds are usually proposed (structure-based drug design approach). It is, however, not clear how to quantify the strength of the TTR/ligand interactions and, crucially, their contribution to the stabilization of the TTR tetramer. Here, we propose a scoring function to quantify the stabilization effect of the ligands based solely on the compactness of the crystal structures of the TTR complexes.

## 2. Results and Discussion

### 2.1. Analysis of Crystal Structures of Selected TTR Mutants

As a proof of concept, the analysis of *CF* was applied to crystal structures of amyloidogenic TTR variants and one variant with protective effects on familial amyloidotic polyneuropathy, taking as reference the wild-type TTR. Selected crystal structures were TTR R104H, a nonamyloidogenic variant with protective clinical effects (pdb id: 1X7T [17]); the amyloidogenic variant TTR Tyr78Phe, which has been shown to react in its soluble tetrameric form with a monoclonal antibody reported to only recognize structures presenting a highly amyloidogenic nature [18] (pdb id: 1X7S [17]); the Val30Met variant, which is associated with the most common form of familial amyloidotic polyneuropathy (FAP) in the Portuguese, Swedish and Japanese populations [19] (pdb id: 1TTC [20]) and the Leu55Pro variant, which is one of the most aggressive variants described so far [21] and has been reported as being implicated in the early onset of FAP (pdb id: 3dk0 [22]). Crystals of these TTR mutants were grown using similar conditions, in acetate or citrate buffer at pH 5.0 to 5.5. Interestingly, other crystallization conditions, leading to a higher amyloidogenic potential such as very low pH and the addition of metal ions, were already used to give insight into the molecular basis of TTR amyloid formation [23,24]. Nuclear magnetic resonance spectroscopy is an alternative technique to investigate the structural details of TTR and has revealed small chemical shifts to the backbone structure of mutated and WT-TTR [25].

**Figure 2:**
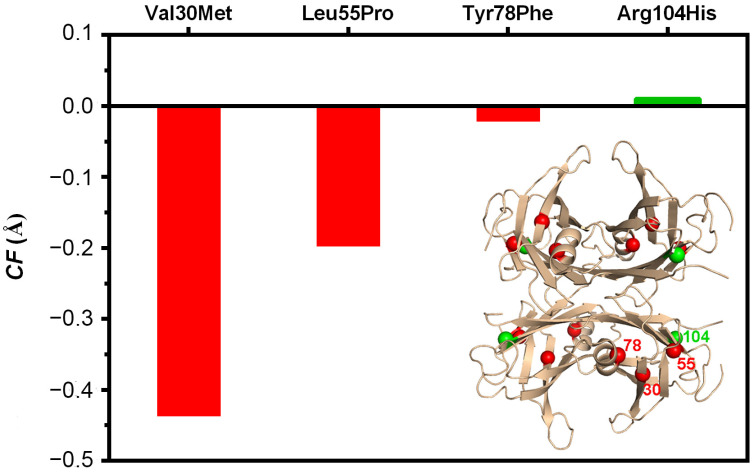
*CF* values (Equation (1)) for the amyloidogenic TTR variants Val30Met, Leu55Pro and Tyr78Phe and for the variant with protective clinical effects, TTR R104H, using the wild-type TTR as reference. Mutations that render proteins less stable also display less compact crystal structures than the reference protein used. Inset: location of the single-point mutations (green protective mutation R104H and red amyloidogenic mutations Val30Met, Leu55Pro and Tyr78Phe).

*CF* values of the amyloidogenic mutants and of the variant with the protective mutation are presented in Figure 2.

The mutations in TTR that lead to an increase in the amyloidogenic propensity are clearly also associated with an increase in the distance between the AB and A’B’ dimers. Interestingly, the mutations of the selected variants (shown in Figure 2) are not located at the AB/A’B’ interface, even though they seem to contribute to the weakness of the interaction between these two symmetry-related dimers and consequently to the relaxation of the quaternary structure of the protein. Most likely, structural differences among TTR mutants are more significant in solution than in the crystalline form, since much experimental evidence points to a breathing mode of TTR in solution [21,26,27,28,29]. Nevertheless, TTR mutations induce subtle but important alterations in their crystal structures (Figure 2). The good predictivity of the *CF* scoring function, when applied to TTR variants, prompted us to extend the analysis towards the crystal structures of complexes of TTR with stabilizing compounds.

### 2.2. Analysis of Crystal Structures of TTR/Kinetic Stabilizers

**Figure 3:**
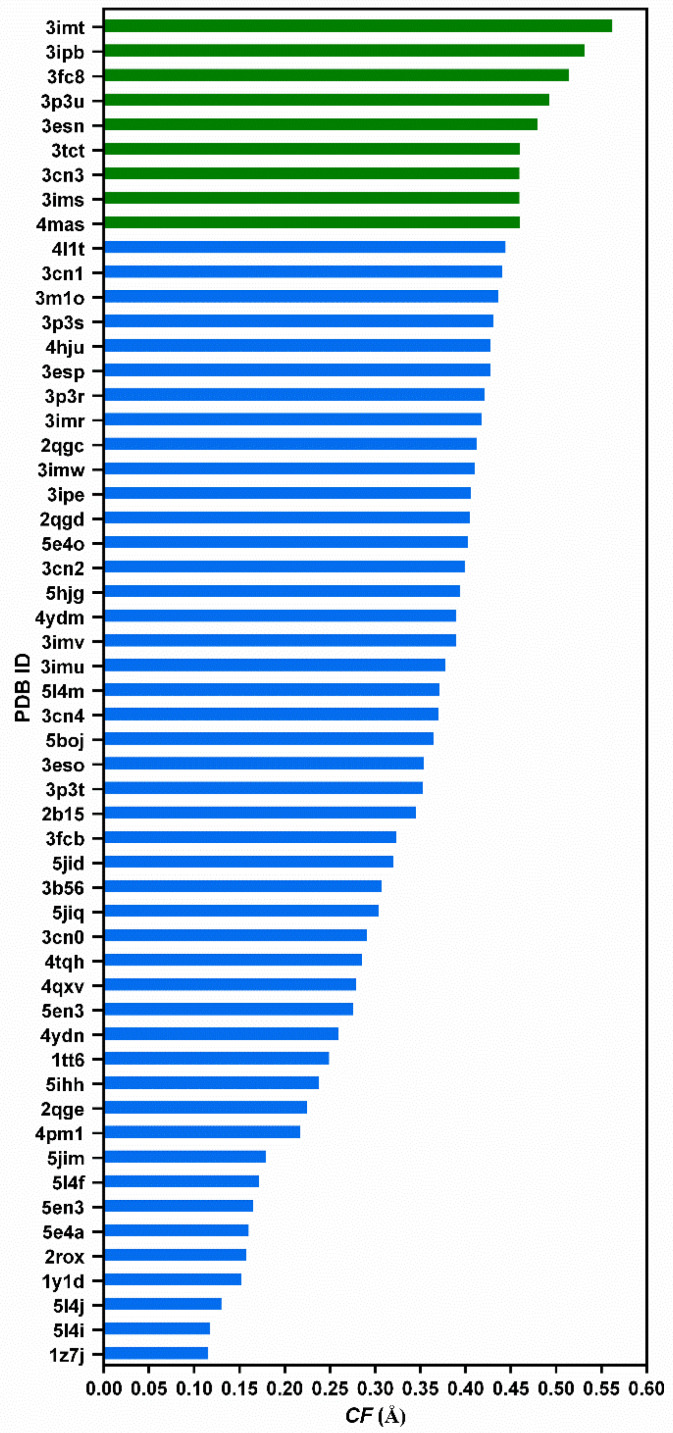
Compactness factor (*CF*) for 55 PDB entries of TTR/kinetic stabilizer complexes, obtained using similar acidic crystallization conditions. The best nine drug candidates (highlighted in green) will be described in detail in Table 1.

The 55 PDB entries of TTR/kinetic stabilizer complexes are listed in Figure 3, together with the calculated *CF* values. They are all positive, which means that all the ligands contribute, on a larger or smaller scale, to the compactness of the tetramer. Interestingly, the structure of TTR with the natural ligand thyroxine (PDB id: 2ROX) has one of the lowest *CF* values, probably due to a “lock-and-key” complementarity with TTR.

It is conceivable that the compactness induced by ligand binding, which, as mentioned earlier, should be more pronounced in solution than in the crystal form, contributes to the observed negative cooperativity in the binding of thyroxine and most kinetic stabilizers. This could result from a slight reduction in the size of the unoccupied binding site due to the binding on the other site. However, other factors such as subtle structural differences between the binding sites could also be contributing. In fact, it has been previously observed that several TTR-binding molecules exhibit a preferential binding to one of the sites [30,31].

**Figure 4:**
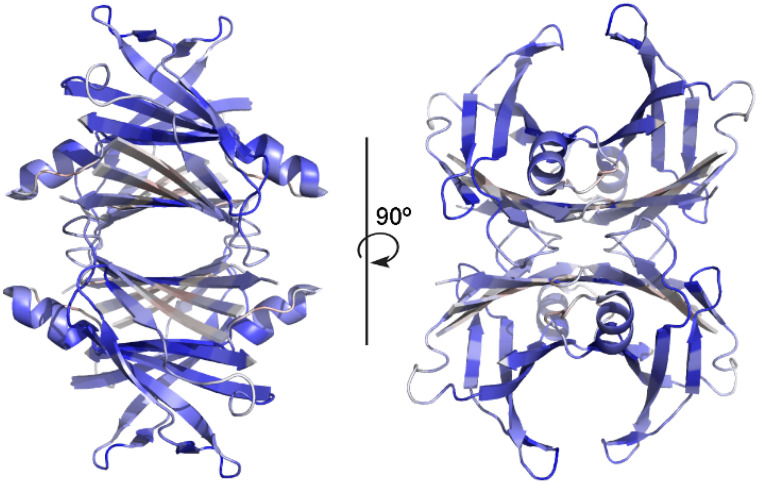
Orthogonal views of TTR highlighting the average deviation by residue ${\left(\partial_{i}\right)}_{ref}-{\left(\partial_{i}\right)}_{Complex}$. Color scheme: red −0.75 Å, grey 0 Å, blue +0.75 Å. Most pronounced deviations induced by the ligand are observed far from the TTR channel.

The average deviations by residue ${\left(\partial_{i}\right)}_{ref}-{\left(\partial_{i}\right)}_{Complex}$ (over the 55 crystal structures analyzed) are presented in Figure 4 (color scheme: red −0.75 Å, grey 0 Å, blue +0.75 Å). It is interesting to notice that the more pronounced deviations upon ligand binding are observed far from the binding site. The β-sheets CBEF from monomers A and B and A’ and B’ that assemble around the central TTR channel display small positive and negative distance deviations to accommodate the guest molecules. It is intriguing that the ligand-induced compactness of the protein is more pronounced in the DAGH β-sheets and in the short α-helix that do not interact directly with the ligand.

The deviations calculated using the CF function, induced by the ligands and by the single-point mutations of the TTR amyloidogenic variants, despite pointing in opposite directions, have similar magnitude (up to 0.5–0.6 Å). These sub-angstrom deviations may look very subtle, but in the case of the amyloidogenic variant, the slight relaxation of the structure has a drastic effect on the stability of the protein and in the development of the disease. On the other hand, one of the compounds listed in Figure 3 has already been approved for treatment of TTR amyloidosis and a few others are in clinical trials; moreover, most of the compounds in Figure 3 were found to stabilize TTR in vitro. Thus, the subtle increase in compaction of the two dimers due to drug binding has a positive effect on the stability of TTR and on the inhibition of the disease progression.

### 2.3. Analysis of the Top-Scored TTR Kinetic Stabilizers

The top-ranked TTR ligands, according to *CF* as calculated by Equation (1), are described in Table 1. Four of the highlighted nine compounds (**1**, **5**, **6b** and **6c**) have two aromatic rings bridged by a short linker. Despite the efforts in the optimization of the linker [32], it does not seem to be a determining feature according to our analysis. Two other compounds have the two aromatic rings directly fused (**3** and **6d**). The 3,5-dibromo-4-hydroxyphenyl substructure is highly favored in the outer thyroxine binding site (**1**, **5**, **6b**, **6c** and **6d** with Br replaced by Cl). Substitutions in 2,6- of the other ring, located in the inner thyroxine binding site, are observed in **5** and **6c**. The *p*-amino substitution in the inner ring induces a bridging hydrogen bond between Ser117/Ser117′ (**1**), while many of the other ligands induce a direct hydrogen bond between Ser117(A)/Ser117(B). Thus, while **1** acts to stabilize the interaction at the weakest AB/A’B’ interface, many of the other ligands stabilize the AA’/BB’ interface. Compound **2** is a long palindromic compound that occupies both binding sites simultaneously. The funnel shape of the binding sites implies that there is a breathing behavior of the protein in solution in order to accommodate the ligand. Other evidence of the relaxation of the protein structure includes the high level of hydrogen/deuterium exchange of residues at the subunit interface [26] and the normal spontaneous intermolecular exchange of TTR subunits [27].

**Table 1:** Top-ranked TTR kinetic stabilizers according to *CF* (Equation (1)).

# PDB	Structure	Description
#1 3imt [33]	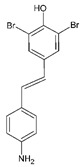	(*E*)-4-(4-aminostyryl)-2,6-dibromophenol: The group of Jeffery Kelly proposed an efficacy score function (Equation (1) from [33]) integrating TTR amyloid inhibition efficacy and plasma TTR binding stoichiometry data. This compound reached the highest score (0.97) over the 92 stilbene and dihydrostilbene analogs synthesized. It exhibits an exceptional human plasma TTR binding stoichiometry (1.93) and a very strong inhibiting effect on fibril formation in vitro. Two binding orientations were observed in the electron density; in the most frequent position, the 3,5-dibromo-4-hydroxyphenyl ring occupies the T4 outer binding subsite, while the p-amino group from the other ring makes bridging hydrogen bonds with Ser117/Ser117′ side chains.
#2 3ipb [29]	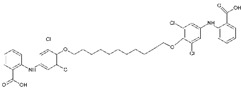	A palindromic bivalent amyloid inhibitor: Ligand binding towards native TTR was irreversible under physiological conditions, stabilizing the tetrameric assembly and inhibiting amyloidogenic aggregation more potently than other known ligands, according to the authors. The binding mode is unique among the tested stabilizers as a single molecule extends over the TTR channel, occupying the two binding sites. The binding of this class of compounds agrees with the existence of a breathing mode of the protein, which could provide a potential route for ligand entry.
#3 3fc8 [34]	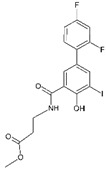	Iododiflunisal-betaAlaOMe: Salicylates look particularly interesting as drug candidates due to their long therapeutic tradition and wide clinical applications. Based on the diflunisal core structure, a salicylate drug with NSAID activity was selected for clinical trials against TTR amyloid diseases, and the authors designed around 40 iodinated derivatives; this one stood out due to the remarkable combined TTR binding vs. aggregation inhibition properties.
#4 3p3u [35]	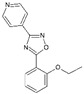	5-(2-ethoxyphenyl)-3-(pyridin-4-yl)-1,2,4-oxadiazole: A total of 33 compounds were investigated according to their TTR amyloidogenesis inhibition properties [35]. Three compounds were able to inhibit aggregation completely under the experimental conditions used. Among the three, this one exhibits the lowest IC50 value.
#5 3esn [36]	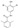	*N*-(3,5-Dibromo-4-hydroxyphenyl)-3,5-dimethyl-4-hydroxybenzamide: Many of the TTR kinetic stabilizers are composed of two aromatic rings and a linker. The authors have previously established optimal structures for one aromatic ring and the linker and, in this work, optimized the second ring substructure (the bottom ring in the figure). They observed that the 2,6-substituted aryls bearing small substituents (like this compound) generate the most potent and selective inhibitors.
#6a 3tct [37]	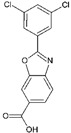	Tafamidis: Most of the TTR kinetic stabilizers emerge from structure-based design studies and thus do not have their pharmacokinetic and pharmacodynamic properties evaluated. Tafamidis was shown by the Kelly group to be a potent and selective transthyretin kinetic stabilizer. It was approved by the European Medicines Agency for the treatment of familial amyloidotic polyneuropathy (FAP) in 2011. Here, we show that tafamidis is also one of the top-ranked compounds for promoting the compactness of TTR.
#6b 3cn3 [32]	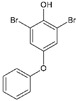	1,3-Dibromo-2-hydroxy-5-phenoxybenzene: In this study, it was shown that the 3,5-dibromo-4-hydroxylphenyl ring strongly prefers to bind in the outer binding site, bridging monomers by making salt bridging interactions with the Lys-15/15′ ε-ammonium groups and maximizing the occupancy of halogen binding pockets (HBPs) 1 and 1′.
#6c 3ims [33]	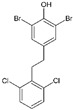	2,6-Dibromo-4-(2,6-dichlorophenethyl)phenol: This compound emerged from the same work as compound #1 and reached an efficacy score of 0.84 and a TTR binding stoichiometry of 1.61. The bromo-substituted ring occupies the outermost part of the binding site (as happens with 6b); the 2,6-dichlorophenyl ring occupies the inner binding subsite, directing the chloride atoms into HBP 3 and 3′.
#6d 4mas	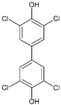	3,3′,5,5′-Tetrachloro-[1,1′-biphenyl]-4,4′diol: The structure of the complex of this compound with TTR was not included in a publication, and therefore, its efficacy is not known. One of the hydroxyl substituents is involved in salt bridge interactions with the Lys-15/15′ side chains, while the chloro atoms occupy the halogen binding pockets (HBPs) 1 and 1′.

Analysis of the structures of the TTR complexes with the top-scored compounds presented in Table 1 shows that the skeleton of the compounds is usually aligned with the C2 crystallographic axis, and the interactions of the ligand substituents with the AB and the A’B’ surfaces are similar.

From the top-ranked compounds, **6a** was approved by the European Medicines Agency for the treatment of familial amyloidotic polyneuropathy (FAP), and **3** is a derivative of diflunisal, an FDA-approved drug that has already been shown to inhibit FAP progression in clinical trials [38]. Pharmacological information from all the others is scarce.

## 3. Materials and Methods

### 3.1. Transthyretin Structural Models

Structural models of TTR determined by X-ray crystallography were obtained from the Protein Data Bank (PDB) [39]. Two groups of TTR atomic coordinates were analyzed. Initially, the structural models of TTR variants with well-known stability were analyzed to confirm the good predictive value of the proposed scoring function; subsequently, a series of crystal structures of transthyretin in complex with amyloid inhibitors was investigated. Most of the structures of TTR complexes deposited in the PDB used acidic crystallization conditions (pH up to 6), usually with a citrate or acetate buffer. There are, however, a smaller number of structures obtained at neutral pH. We found that the two groups could not be directly compared because there were systematic deviations in the atomic coordinates due to the crystallization pH that should not be attributed to a ligand-binding effect (it is well known that the pH affects the stability of TTR and that the formation of amyloid fibrils can be induced in strongly acidic conditions); so, the analysis was restricted to the ones obtained at an acidic pH. A total of 55 PDB entries of TTR/kinetic stabilizer complexes were examined [10,29,32,33,34,35,36,37,40,41,42,43,44,45,46,47,48,49,50,51,52,53,54,55]. The ligand position usually lies on the crystallographic axis, which means that there are two symmetry-related positions in each binding site. In some models, two positions are refined in each binding site of the asymmetric unit. The occupation of the atoms of the ligand is typically set to give full saturation (one molecule per binding site) due to the usual high affinity between the protein and the drug candidates. All the models were compared to a reference crystal structure of the apo-TTR. An analysis of several apo-TTR structures available in the PDB showed negligible deviations between these models when compared to the deviations obtained towards the TTR/stabilizer complexes. The high-resolution model of the apo-TTR pdb id: 2QGB [49] was used as a reference.

### 3.2. The Scoring Function

**Figure 5:**
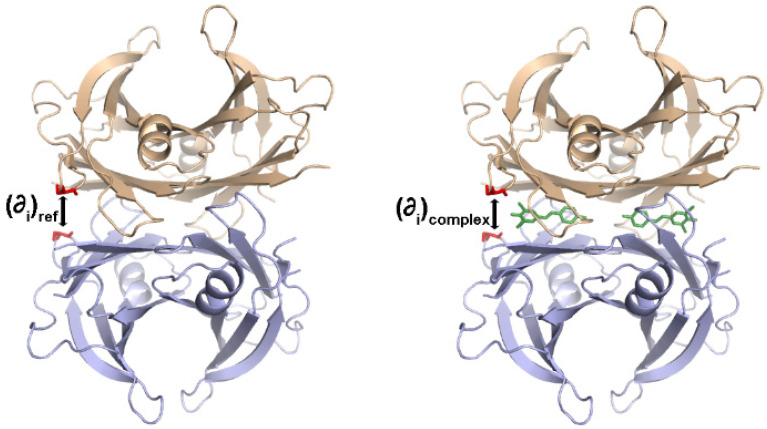
Crystal structures of WT-TTR in the Apo-form (**left**) and in complex with a TTR kinetic stabilizer (**right**). Monomers AB in salmon and A’B’ in blue. Distances between the $C_{\alpha }$ of the symmetry-related residues *i* and *i*’ highlighted in red are illustrated.

The energetically weaker interface of TTR is formed by the two symmetry-related dimers AB/A’B’ (Figure 1). TTR kinetic stabilizers bind in the T4 binding sites, establishing hydrophobic and/or polar interactions with AB and A’B’ simultaneously, which presumably inhibits the dissociation of the dimers. We hypothesize that the stabilizing effect of the ligands can be correlated with how much they can bring the dimers AB and A’B’ close together. In fact, the increased contact between dimers due to mutation of the residues lining the interdimer cavity was already observed to increase TTR stability [56]. Hence, the deviation in the interdimer distance (AB and A’B’) induced by the binding of the kinetic stabilizers was calculated; the compactness factor (*CF*) was defined as follows:

(1) $$CF=\sum_{i}\frac{w_{B,i}\left[{\left(\partial_{i}\right)}_{ref}-{\left(\partial_{i}\right)}_{Complex}\right]}{N},$$

where $\partial_{i}$ stands for the distances between $C_{\alpha }$ atoms of residues *i* and *i*’ and ${\left(\partial_{i}\right)}_{ref}-{\left(\partial_{i}\right)}_{Complex}$ measures the deviation of $\partial_{i}$ between a reference model (an apo-TTR pdb id: 2QGB) and the model of the complex (illustrated in Figure 5). The local mobility of $C_{\alpha }$ in TTR was considered by taking the weighting factor $w_{B,i}=\bar{B}/B_{i}$, where $\bar{B}$ and $B_{i}$ are the average and the residue *i* temperature factors of $C_{\alpha }$ of the reference structure. Moreover, the *N*- and *C*-terminals of the two chains in the asymmetric unit are usually too flexible to see, and typically, the crystal models comprise residues 11–124. Each chain has 127 residues.

According to Equation (1), structural models more compact than the reference will give positive *CF* values, while negative *CF* will be obtained for structures that are less tight than the apo-TTR and, presumably, also less stable structures.

## 4. Conclusions

Here, a scoring function based on interdimer AB/A’B’ Cα distances (*CF*, compactness factor) was proposed to easily rank TTR kinetic stabilizers using X-ray crystallography data. A total of 55 TTR kinetic stabilizers, which were cocrystallized with TTR under similar acidic conditions and whose structures were deposited in the PDB, were analyzed. *CF* was calculated using Equation (1), and the TTR kinetic stabilizers were ranked accordingly (Table 1). Positive *CF* values show that kinetic stabilizers induce compactness of TTR in the crystal form.

Top-ranked compounds (Table 1) were also highly ranked by the authors in their original publications using other criteria, such as TTR amyloid inhibition efficacy and plasma TTR binding stoichiometry, providing evidence of a positive correlation between an increase in *CF* and standard biochemical assays used to evaluate the drug candidates. Moreover, the proposed *CF* score appears to be also valid when applied to TTR mutations with recognized amyloidogenic properties (in which a *CF* decrease is observed). This compacting effect should be more pronounced at physiological conditions, as much experimental evidence points to a breathing behavior of TTR in solution. The application of this scoring method to select a compound must, however, be complemented with biochemical studies, such as the ligand affinity towards TTR (usually cocrystallization experiments are performed using a significant excess of the ligand) and selectivity and pharmacokinetics towards TTR in plasma. Selectivity towards TTR in plasma samples is crucial to investigate because the hydrophobic character of most of the drug candidates drives its binding to the TTR channel in in vitro assays with pure TTR samples.

As TTR is routinely cocrystallized in the presence of ligands of interest, our function can be a good method to use that data in other ways than the traditional assessment of binding mode and allow an initial selection of the better anti-TTR amyloidosis candidates.

## Data Availability

Data are contained within the article.
